# Supplementary material for: Profiles and interactions of gut microbiome and intestinal microRNAs in pediatric Crohn’s disease
Source: mSystems. 2024 Aug 16;9(9):e00783-24. doi: 10.1128/msystems.00783-24 (PMC11406922; doi:10.1128/msystems.00783-24)
Supplement: Supplemental File — Figures S1 to S3, Table S1, and Table S5. [file msystems.00783-24-s0001.docx]

**Supplemental Legends**


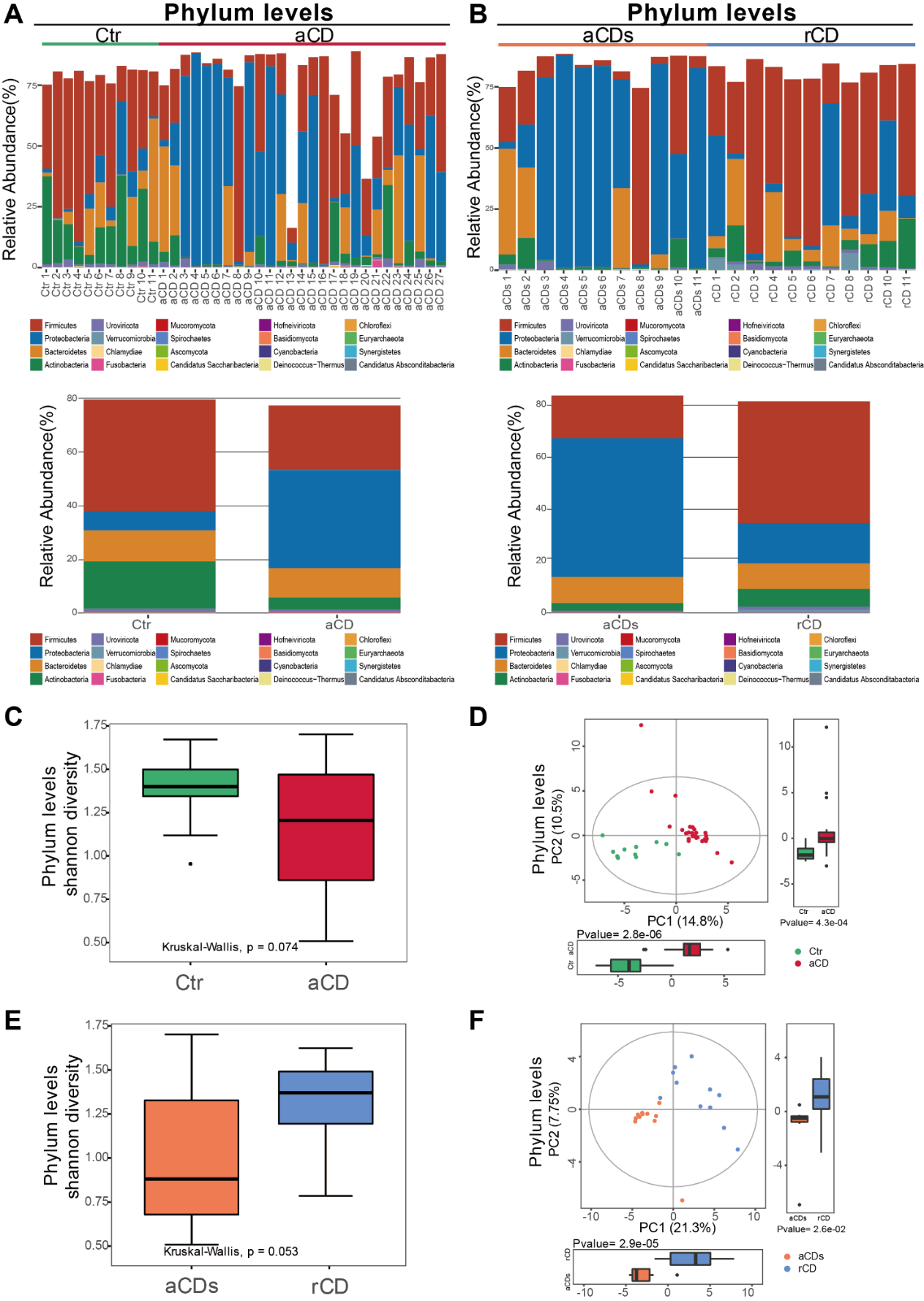


**Fig.s1 Differential GM profiles in four groups at the phylum level**

Relative abundance of the 20 most abundant microbial and mean community composition of each microbial state at the phylum level (A-B) for each group. (C, E) Shannon diversity differs significantly across microbial states at the phylum level for each group. (D, F) Principal coordinate analysis (PCoA) profile of microbial diversity illustrates that gut bacterial communities are compositionally distinct at the phylum level for each group.


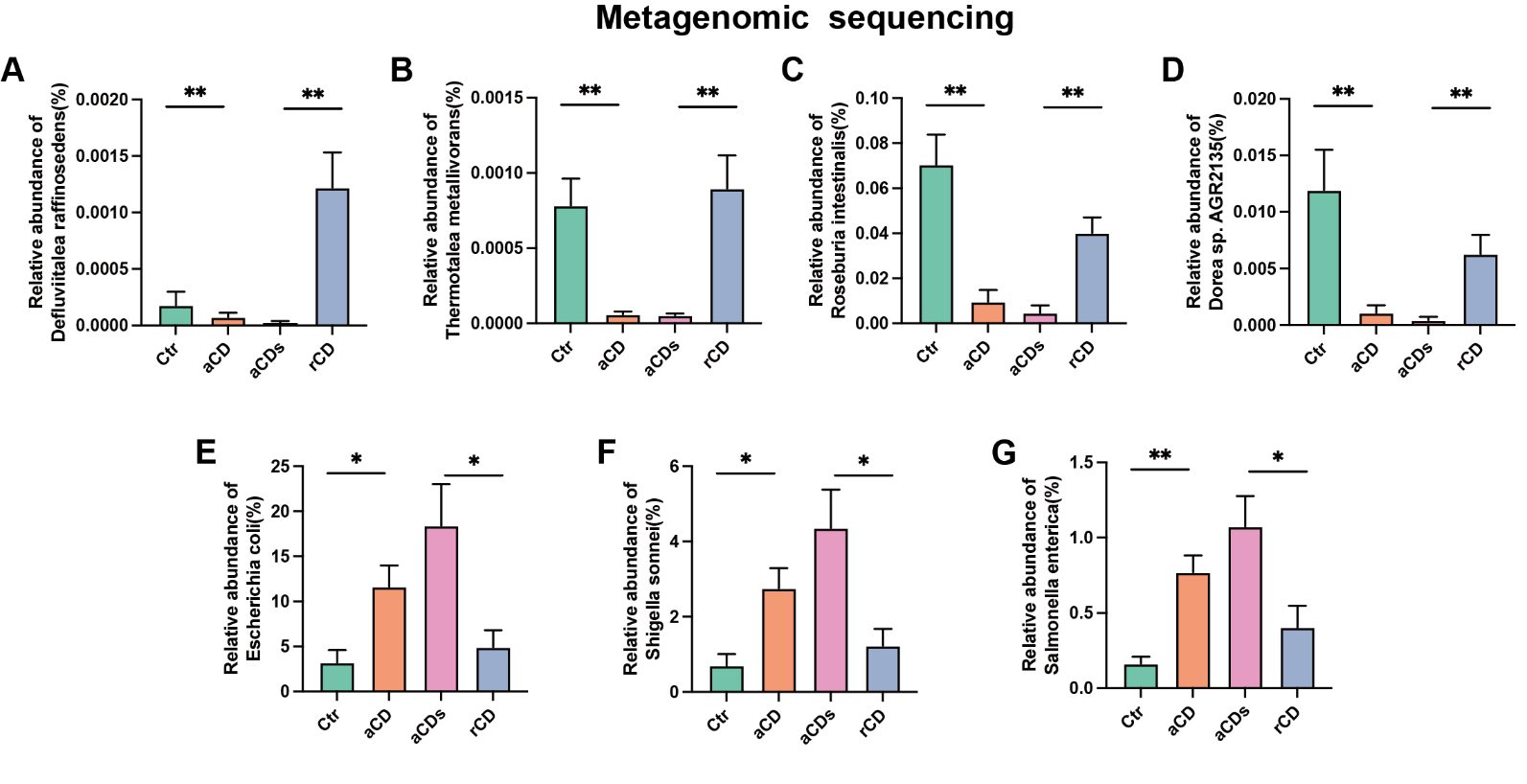


**Fig.s2 Relative proportion of seven key bacteria among groups**

(A-G) The relative proportion of *Defluviitalea raffinosedens*, *Thermotalea metallivorans*, *Roseburia intestinalis*, *Dorea sp. AGR2135*, *Escherichia coli*, *Shigella sonnei* and *Salmonella enterica* in four groups from metagenomic sequencing data. *: p-value < 0.05, **: p-value < 0.01.

**
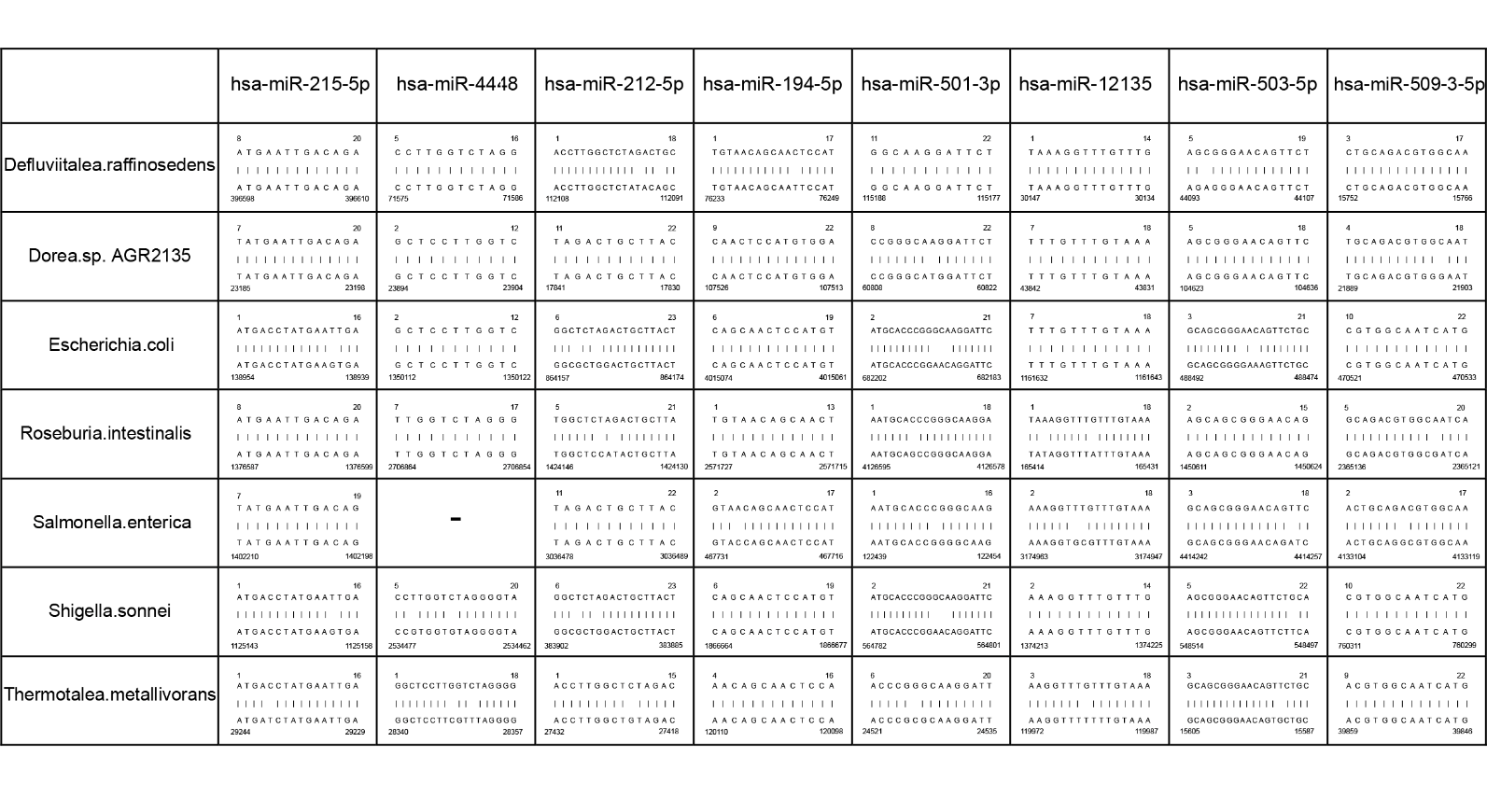
**

**Fig.s3 Schematic diagram of the putative binding sites of the seed sequence of miRNAs in the genes of gut microbiome of the minimal e-value**

Predicted consequential pairing of target regions were shown in the bottom of blank and miRNA seed sequences were listed in the top of blank.

**Table s 1. Comparation of the clinical parameters of aCD and aCDs groups.**

|  | aCD | aCDS | P value |
| --- | --- | --- | --- |
| n | 27 | 11 |  |
| Age(yr) | 13.09±1.46 | 13.17±1.20 | 0.87 |
| Males/Females | 18/9 | 10/1 | 0.12 |
| HFA z-score | 0.14±1.16 | 0.41±1.12 | 0.53 |
| BMI z-score | -1.51±1.87 | -0.87±1.92 | 0.36 |
| WBC(10^9/L) | 9.72±3.79 | 9.49±3.89 | 0.87 |
| NE(10^9/L) | 6.55±2.93 | 6.08±3.15 | 0.67 |
| LY(10^9/L) | 2.14±1.09 | 2.21±0.78 | 0.50 |
| RBC(10^12/L) | 4.37±0.64 | 4.56±0.64 | 0.44 |
| HB(g/L) | 101.26±27.95 | 107.82±24.60 | 0.48 |
| Plt(10^9/L) | 455.04±110.37 | 466.73±116.72 | 0.78 |
| TBA(umol/L) | 6.01±4.82 | 5.56±2.47 | 0.77 |
| ALT | 11.11±7.95 | 9.82±4.42 | 0.62 |
| AST | 17.19±7.31 | 17.82±5.98 | 0.80 |
| Cre | 53.48±16.53 | 55.45±16.75 | 0.74 |

aCD: patients who achieved the PCDAI≥10 points before induction therapy were defined as active CD; aCDs: for those remission CD before induction therapy were defined as active CD (aCDs) (active CD subgroup); HFA: height for age; BMI: body mass index; WBC: white blood cell; NE: neutrophil; LY: lymphocyte; RBC: red blood cell; HB: hemoglobin; Plt: blood platelet; TBA: total biliary acid; ALT: alanine transaminase; AST: aspartate transaminase; Cre: creatinine

**Table s5. The p-value value and R score of miRNAs, PCDAI, CDEIS score and calprotectin. R: spearman's rank correlation coefficient.**

|  | PCDAI | |  | Calprotectin | |  | CDEIS | |
| --- | --- | --- | --- | --- | --- | --- | --- | --- |
|  | R | P value |  | R | P value |  | R | P value |
| hsa-miR-12135 | -0.5038 | 0.0013 |  | -0.3647 | 0.0250 |  | -0.3470 | 0.0328 |
| hsa-miR-509-3-5p | -0.4862 | 0.0020 |  | -0.3259 | 0.0458 |  | -0.3665 | 0.0069 |
| hsa-miR-212-5p | 0.4257 | 0.0077 |  | 0.6319 | 0.0001 |  | 0.4306 | 0.0275 |
| hsa-miR-4448 | 0.3622 | 0.0254 |  | 0.5201 | 0.0010 |  | 0.3577 | 0.0363 |
| hsa-miR-501-3p | 0.3255 | 0.0461 |  | 0.6288 | 0.0001 |  | 0.3407 | 0.0131 |
| hsa-miR-503-5p | 0.5007 | 0.0014 |  | 0.4728 | 0.0030 |  | 0.3988 | 0.0236 |
